# Sulfonate-Conjugated Polyelectrolytes as Anode Interfacial Layers in Inverted Organic Solar Cells

**DOI:** 10.3390/molecules26030763

**Published:** 2021-02-02

**Authors:** Elisa Lassi, Benedetta Maria Squeo, Roberto Sorrentino, Guido Scavia, Simona Mrakic-Sposta, Maristella Gussoni, Barbara Vercelli, Francesco Galeotti, Mariacecilia Pasini, Silvia Luzzati

**Affiliations:** 1 Institute of Chemical Sciences and Technologies “G. Natta ”-SCITEC, National Research Council, CNR-SCITEC, via Corti 12, 20133 Milan, Italy; lassi@ismac.cnr.it (E.L.); benedetta.squeo@scitec.cnr.it (B.M.S.); roberto.sorrentino@scitec.cnr.it (R.S.); guido.scavia@scitec.cnr.it (G.S.); maristella.gussoni@unimi.it (M.G.); francesco.galeotti@scitec.cnr.it (F.G.); 2 Institute of Clinical Physiology, National Research Council, CNR-IFC, Piazza Ospedale Maggiore 3, 20162 Milan, Italy; simona.mrakicsposta@cnr.it; 3 Institute of Condensed Matter Chemistry and Technologies for Energy, National Research Council, CNR-ICMATE, Via Roberto Cozzi 53, 20125 Milan, Italy; barbara.vercelli@cnr.it

**Keywords:** conjugated polyelectrolytes, inverted organic solar cells, anode interfacial layers

## Abstract

Conjugated polymers with ionic pendant groups (CPEs) are receiving increasing attention as solution-processed interfacial materials for organic solar cells (OSCs). Various anionic CPEs have been successfully used, on top of ITO (Indium Tin Oxide) electrodes, as solution-processed anode interlayers (AILs) for conventional devices with direct geometry. However, the development of CPE AILs for OSC devices with inverted geometry is an important topic that still needs to be addressed. Here, we have designed three anionic CPEs bearing alkyl-potassium-sulfonate side chains. Their functional behavior as anode interlayers has been investigated in P3HT:PC_61_BM (poly(3-hexylthiophene): [6,6]-phenyl C61 butyric acid methyl ester) devices with an inverted geometry, using a hole collecting silver electrode evaporated on top. Our results reveal that to obtain effective anode modification, the CPEs’ conjugated backbone has to be tailored to grant self-doping and to have a good energy-level match with the photoactive layer. Furthermore, the sulfonate moieties not only ensure the solubility in polar orthogonal solvents, induce self-doping via a right choice of the conjugated backbone, but also play a role in the gaining of hole selectivity of the top silver electrode.

## 1. Introduction

The use of interfacial layer materials to improve the charge selectivity and minimize the energy barrier of electrodes plays a central role in promoting their performance and stability in organic electronic devices, such as organic solar cells (OSCs) [1,2,3]. In view of the technological need to attain fully solution-processed devices, most of the attention has been addressed in the search of efficient interfacial materials with good solubility in polar solvents, included water, which allows deposition from orthogonal solvents to the active layers [4,5,6]. Conjugated polyelectrolytes (CPEs), composed of a conjugated backbone with side-chains bearing ionic functional groups, have emerged as a promising class of interfacial materials with their proven ability to improving photovoltaic (PV) performances through solution processing [7,8,9,10]. CPEs combine several advantages including: solubility in aqueous/alcoholic solvents, orthogonality to organic solvents used for the deposition of the active layers, robust film formation, and chemical flexibility in tailoring both the conjugated backbone as well as the polar/ionic lateral functionalities. Most of the CPEs developed so far are effective in reducing the electrode work function [11,12,13] and might behave as electron transport layers [14,15]. Therefore there is a huge library of CPEs in the literature that have been investigated as cathode interfacial layers to facilitate the electron collection at the cathode in OSCs [16,17,18,19,20,21]. There is, however, also a growing number of CPEs that have been reported to behave as promising anode interfacial layers to facilitate hole extraction at the ITO electrode [22,23,24,25,26,27,28,29,30]. These CPEs were demonstrated to be a valid alternative to the common PEDOT:PSS, not only for the good PV performances, but also because most of these materials are pH-neutral, which is an advantage to avoid possible device instabilities induced by the acidic nature of PEDOT:PSS [31,32].

From the studies so far, some guidelines have been reported for the design of the CPEs where their application as anode modifiers were highlighted. It was shown that upon oxidative p-doping, a fluorene-based CPE with anionic sulfonate side groups deposited on top of an ITO electrode behaved as an efficient AIL in OSCs [23]. The p-doping not only favored the hole transport through the inter-layer, but it was shown to be a viable tool to enhance the ITO anode work function [23]. When combining anionic sulfonate side groups to polymer chains containing more electron rich moieties than fluorene, p-type self-doping effects can occur [33,34]. Self-doping was found to induce similar beneficial effects for the hole transport and ITO anode work function modifications [22,24,35], and it was identified as an important characteristic to achieve efficient AIL materials [24,25,26,28,36].

In spite of the successful incorporation of various CPEs as anode modifiers into devices, mainly devices with direct geometry were reported. Nevertheless, the devices with inverted geometry are better suited to envisage the scale-up towards industrial compatible fabrication processes. Therefore, the application of CPE materials as anode modifiers in inverted OSC devices is an important topic to be addressed.

The capability of a CPE to engineer the anode interface, established in direct geometry, is not necessarily transposable to inverted devices. For example, most of the efficient CPE anode modifiers reported in the literature are quite hydrophilic and need water to be dissolved. This can be an issue for inverted geometry, where the CPE film is deposited on top of the highly hydrophobic active layer surface. This implies that the tailoring of the chemical structure of CPEs to improve their reaction with the active layer is important when considering the inverted device geometry. Another aspect that may vary with the device geometry is the interaction between the interlayer and the electrode which is not necessarily identical when depositing the interlayer on top of the electrode, as is the case for direct geometry, or when evaporating a metal electrode on top of the interlayer, as is the case for inverted geometry.

**Figure 1:**
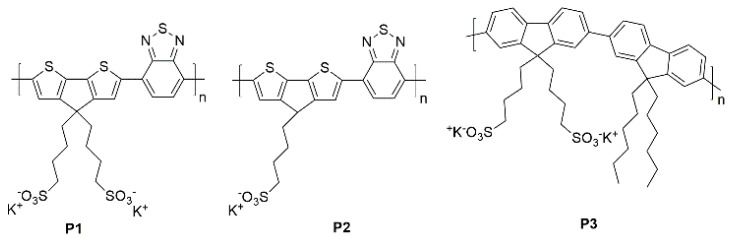
Chemical structure of the synthesized CPEs.

In this work, we have designed three anionic conjugated copolymers bearing alkyl-potassium sulfonate side groups, namely **P1**, **P2**, and **P3** (see Figure 1), and their application as anode interfacial layers for OSCS devices with inverted geometry have been investigated. The devices are prepared using standard P3HT:PC_61_BM as an active layer. We have started with a well-known CPE, **P1** [34] in Figure 1, which is reported to have good results when applied in OSCs devices with direct geometry [22,27,31]. **P1** is a pH-neutral CPE that showed self-doping characteristics, in particular in the presence of a proton source, good hole conductivity comparable to PEDOT:PSS, and effective engineering of the ITO anode work function [34,37]. **P1** is hydrophilic owing to the high number of polar groups per monomeric unit and needs water to be dissolved. In order to grant self-doping characteristics, but obtain an alcohol-processable material which guarantees better wettability of the active layer, we have designed a novel copolymer, **P2**, Figure 1, with the same backbone and type of alkyl- sulfonate substituents of **P1** but with a reduced number of side chains per monomeric unit. Finally, for comparison, we have prepared and tested **P3**, in Figure 1, a CPE bearing similar potassium sulfonate alkyl groups as **P1** and **P2** but with a different conjugated backbone based on a fluorenic unit, known to be un-fitted for self-doping [34,38,39,40]. In the **P3** copolymer, the side chains have been tailored to favor the interaction with the active layer by alternating alkyl-potassium sulfonate and non-polar alkyl side chains. By comparing the results obtained with the three CPEs we tried to elucidate the role that pendant sulfonate groups play in the AIL functionality of this class of polymers, demonstrating that sulfonate anionic CPEs could be a valid approach to obtain solution-processable anode interlayers for inverted OSCs devices.

## 2. Experimental

### 2.1. Synthetic Methods

General information for synthesis: All glassware was oven-dried. Unless specifically mentioned, all chemicals are commercially available and were used as received. The dialysis membrane (MWCO: 3500–5000 Da) was purchased from Membrane Filtration Products Inc. ^1^H-NMR spectra were recorded at 600 MHz in D_2_O.

*4-Bis-potassium butanylsulfonate-4H-cyclopenta-[2,1-b;3,4-b’]-dithiophene* (**1**): 4H-cyclopenta-[2,1-b;3,4-b’]-dithiophene (CPDT, 670 mg, 3.76 mmol, 1.0 equivalent), and tetrabutylammonium bromide (60.6 mg, 0.188 mmol, 0.05 equivalent), were dissolved in anhydrous DMSO (18.4 mL), and the solution was degassed by bubbling Ar for 5 min. 50% KOH in H_2_O (4.2 g) was added via syringe, followed by the addition of 1,4-butanesultone (924 µL, 9.02 mmol, 2.4 equivalent). After stirring at room temperature for 3 h, the reaction mixture was poured into acetone (100 mL) and the yellowish precipitate was collected by filtration and washed with acetone. The crude was used in the next step without further purification. ^1^H-NMR (D_2_O) δ: 7.35 (d, *J* = 4.8 Hz, 2H), 7.14 (d, *J* = 4.8 Hz, 2H), 2.72 (t, *J* = 8.1 Hz, 4H), 1.98 (t, *J* = 7.8 Hz, 4H), 1.55 (m, broad, 4H), 0.98 (m, broad,4H).

*2,6-Dibromo-4-bis-potassium butanylsulfonate-4H-cyclopenta-[2,1-b;3,4-b′]-dithiophene* (**2**): The crude product 1 was suspended in DMF (15 mL), and H_2_O (~2 mL) was added while stirring until dissolved. NBS (1.67 g, 9.4 mmol, 2.5 equivalent) was added in dark conditions by shielding the flask with aluminum foil. The brown solution was stirred at room temperature for 1 h, and poured into acetone. The yellowish precipitate was collected by filtration, and washed with acetone (2 g, 80% yield). ^1^H-NMR (D_2_O) δ: 7.03 (s, 2H), 2.6 (m, broad, 4H), 1.69 (m, broad, 4H), 1.43 (m, broad, 4H), 0.77 (m, broad, 4H).

Polymer **P1**: A mixture of compound **2** (79 mg, 0.115 mol, 1 equivalent), 2,1,3-Benzothiadiazole-4,7-bis(boronic acid pinacol ester) (45 mg, 0.115 mmol, 1 equivalent), and tetrakis(triphenylphosphine)palladium(**0**) (Pd(PPh_3_)_4_) (2.6 mg, 2% mol) was added in a pre-degassed Schlenk flask, followed by three vacuum/nitrogen cycles. Then degassed DMF (1 mL) and degassed potassium carbonate aqueous solution (0.25 mL) were added. The mixture was stirred at 110 °C for 3 h. The reaction mixture was poured in acetone and the dark blue precipitate was collected by filtration and washed with copious amounts of acetone. The precipitate was all dissolved in deionized H_2_O and transferred into a dialysis tube (MWCO: 3500–5000). The dialysis tube was placed in a large beaker with H_2_O stirring for 3 days, and the H_2_O was changed every 12 h. Evaporation of the H_2_O provided the title product, a dark blue solid (55 mg, 72%), after drying under vacuum overnight. The NMR of the polymer in D_2_O showed only non-informative broad peaks, due to the presence of paramagnetic radical cations [34] (see Scheme S1 in Supplementary Materials).

*Synthesis of 4-Potassium butanylsulfonate-4H-cyclopenta-[2,1-b;3,4-b’]-dithiophene* (**3**): 4H-cyclopenta-[2,1-b;3,4-b’]-dithiophene (CPDT, 300 mg, 1.68 mmol, 1.0 equivalent) and tetrabutylammonium bromide (27 mg, 0.084 mmol, 0.05 equivalent) were dissolved in anhydrous DMSO (8.2 mL), and the solution was degassed by bubbling with Ar for 5 min. 50% KOH in H_2_O (1.8 g) was added via syringe, followed by the addition of 1,4-butanesultone (924 µL, 9.02 mmol, 1.2 equivalent). After stirring at room temperature for 3 h, the reaction mixture was poured into acetone (50 mL) and the yellowish precipitate was collected by filtration and washed with acetone. The crude was used in the next step without further purification. ^1^H-NMR (D_2_O) δ: 7.05 (d, *J* = 4.8 Hz, 2H), 6.95 (d, *J* = 2.9 Hz, 2H), 3.42(t, *J* = 6.9 Hz, 1H) 2.73 (t, *J* = 7.8 Hz, 2H), 1.63 (m, broad, 2H), 1.56 (m, broad, 2H), 1.3 (m, broad,2H).

*Synthesis of 2,6-Dibromo-4-potassium butanylsulfonate-4H-cyclopenta-[2,1-b;3,4-b’]-dithiophene* (**4**): The crude product **3** was suspended in DMF (6.7 mL), and H_2_O (~1 mL) was added while stirring until dissolved. NBS (747 g, 4.2 mmol, 2.5 equivalent) was added in dark conditions by shielding the flask with aluminum foil. The brown solution was stirred at room temperature for 1 h and poured into acetone. The yellowish precipitate was collected by filtration, and washed with acetone (640 mg, 75% yield). ^1^H-NMR (D_2_O) δ: 7.23 (s, 2H), 3.74 (t, broad, 1H), 2.80 (m, broad, 2H), 2.60–2.50 (m, broad, 2H), 1.85–1.80 (m, broad, 2H), 1.55–1.45 (m, broad, 2H).

Synthesis of polymer **P2**: A mixture of compound **3** (117 mg, 0.230 mol, 1 equivalent), 2,1,3-Benzothiadiazole-4,7-bis(boronic acid pinacol ester) (89 mg, 0.230 mmol, 1 equivalent), and tetrakis(triphenylphosphine)palladium(**0**) (Pd(PPh_3_)_4_) (5.2 mg, 2% mol) was added in a pre-degassed Schlenk flask, followed by three vacuum/nitrogen cycles. Then degassed DMF (2.2 mL) and degassed potassium carbonate aqueous solution (0.55 mL) were added. The mixture was stirred at 110 °C for 3 h. The reaction mixture was poured in acetone and the dark blue precipitate was collected by filtration and washed with copious amounts of acetone. The precipitate was all dissolved in deionized H_2_O and transferred into a dialysis tube (MWCO: 3500–5000). The dialysis tube was placed in a large beaker with H_2_O stirring for 3 days, and the H_2_O was changed every 12 h. Evaporation of H_2_O provided the title product, a dark blue solid (65 mg, 60%), after drying under vacuum overnight. The NMR of the polymer in D_2_O showed only non-informative broad peaks, due to the presence of paramagnetic radical cations (see Scheme S2 in Supplementary Materials) [34].

### 2.2. Device Fabrication and Photovoltaic Characterization

Direct geometry devices fabrication: Solar cells were assembled with the conventional structure Glass/ITO/PEDOT:PSS or AIL/P3HT:PC_61_BM/Ag. Glass ITO (Kintec, Hong Kong) 15 Ω/sq substrates were mechanically cleaned with peeling tape and paper with acetone and then were washed in a sonic bath at 50 °C for 10 min sequentially with water, acetone, and isopropanol. After drying with compressed nitrogen flow, 10 min plasma treatment in the air was used to enhance the ITO wettability for the next deposition. PEDOT:PSS (Al VP 8030 from Heraus, Hanau, Germany) was filtered on a 0.45 μm nylon filter and spin-coated in the air at 2500 rpm for 50 s. **P1** (5 mg/mL in H_2_O:MeOH 1:1), **P2,** and **P3** (1 mg/mL in EtOH) were spin-coated at 2000 rpm and 4000 rpm for 60 s. Finally, the substrates were stored in a glovebox and annealed at 110 °C for 10 min. The device assembly was then performed in the glovebox. The active layer was composed of a blend dissolved at 1:0.8 wt/wt of P3HT:PC61BM solution in 1,2-dichlorobenzene at a total concentration of 27 mg/mL. The P3HT was purchased from Plextronics (Pittsburgh, PA, USA, Plexcore 0S2100, Mn: 62602; Mw: 119010, 99% regioregularity) and PC_61_BM (99.5 % purity) was purchased from Solenne BV. The solution was stirred for 12 h on a hotplate in a glovebox at 60 °C. The active layer was spin-coated from the warm solution at 1000 rpm for 60 s, which resulted in a thickness of 130 nm; then, the active layer film was slow dried under a glass petri dish for 1 h. Finally, a 100 nm-thick aluminum electrode was evaporated on the top of the device through a shadow mask under a pressure of 2 × 10^−6^ mbar. The deposition rate was 0.5 nm/s. There were six devices on a single substrate, each with an active area of 6.1 mm^2^.

Inverted geometry devices fabrication: Solar cells were assembled with the conventional structure Glass/ITO/PEIE/active layer/AIL/Ag. Glass ITO (Kintec, Hong Kong) 15 Ω/sq substrates were mechanically cleaned with peeling tape and paper with acetone and then were washed in a sonic bath at 50 °C for 10 min sequentially with water, acetone, and isopropanol. After drying with compressed nitrogen flow, ITO was treated under UV light from a solar simulator for 20 s to remove O_2_ species adsorbed on its surface. Polyethylenimine ethoxylated (PEIE) 0.4% *w/w* in 2-Metoxyethanole solution was spin-coated in N_2_ at 5000 rpm for 60 s. PEIE film was then washed with H_2_O to remove excess polymers (200 µL were deposited for 10 s on substrate and then removed at 4000 rpm for 60 s). The P3HT:PC_61_BM active layer was prepared upon blending the two components at a 1:0.8 weight ratio in 1,2-dichlorobenzene, at a total concentration of 27 mg/mL. The solution was stirred for 12 h on a hotplate in glovebox at 60 °C; the active layer was spin-coated from the warm solution at 1000 rpm for 60 s, which results in a thickness of 130 nm; the films were then covered with a glass petri dish to perform slow drying for an hour and then treated at 120 °C for 10 min. The PTB7-Th was purchased from Cal-Os (batch number 6) and PC_71_BM from Solenne BV (99.5% purity). The PTB7-Th:PC71BM active layer was prepared by dissolving the two components at a 1:1.5 weight ratio in chlorobenzene, with a solute concentration of 25 mg/mL. This solution was stirred overnight at 65 °C. Next, 10 min after adding 2% *v/v* of 4-methoxybenzaldehyde to the solution, the blend was spin coated at 1200 rpm, permitting the obtaining of films with thickness of 100 nm. The samples were left at room temperature for 10 min and then annealed for 20 min at 60 °C. The AILs were deposited on top of the active layers by dissolving **P2** or **P3** in ethanol at a concentration of 1 mg/mL, and 20 μL of this solution was dropped on a device rotating at 4000 rpm for 60 s. **P1** was dissolved in H_2_O:Methanol (1:1) at a concentration of 5 mg/mL, and 20μL of this solution was dropped on a device rotating at 4000 rpm for 60 s. Finally, a 100 nm-thick silver electrode was evaporated on the top of the device through a shadow mask under a pressure of 2 × 10^−6^ mbar. The deposition rate was 0.3 nm/s. A device without any AIL and a device with an evaporate MoOx AIL were prepared for comparison. The thickness of the MoOx layer was 10 nm, achieved with a deposition rate of 0.1 nm/s. There were six devices on a single substrate, each with an active area of 6.1 mm^2^.

Device characterization and measurements: The devices were characterized through current density–voltage and external quantum efficiency characterization. Current density–voltage measurements were performed directly in the glovebox where the solar cells were assembled, with a Keithley 2602 source meter, under an AM 1.5G solar simulator (ABET 2000). The incident power, measured with a calibrated photodiode (Si cell + KG5 filter), was 100 mW/cm^2^. The EQE spectral responses were recorded by dispersing an Xe lamp through a monochromator, using an Si solar cell with a calibrated spectral response to measure the incident light power intensity at each wavelength. The devices were taken outside the glovebox for the EQE measurements, after mounting them on a sealed cell to avoid moisture and oxygen exposure. For the aging measurement, the devices were stored in air and measured in glovebox.

## 3. Results and Discussion

The chemical structures of the three copolymers **P1**, **P2**, and **P3**, are shown in Figure 1. The polymers were synthesized via Suzuki cross-coupling and their purifications were performed according to the literature. The selected experimental procedures were chosen according to the literature [41,42]; **P1** was prepared according to the procedure described by Mai et al. [34] starting from the commercially available 2,1,3-Benzothiadiazole-4,7-bis(boronic acid pinacol ester) and 2,6-dibromo-4,4-bis-potassium butanylsulfonate-4H-cyclopenta-[2,1-b;3,4-b’]-dithiophene. The same procedure was applied to **P2**, starting from the newly synthesized 2,6-dibromo-4-potassium butanylsulfonate-4H-cyclopenta-[2,1-b;3,4-b’]-dithiophene, while **P3** was synthetized according to the literature [39,40] (synthesis details and characterizations are provided in the Supplementary Materials). GPC measurements confirmed the structure of the synthetized polymers, however they only provided an indicative estimation of the real value of the molecular weight due to solubility problems and structural difference with the polystyrene used as standard [43].

Cyclic voltammetry and optical spectroscopy were used to assess the electronic properties of the copolymers and to estimate their HOMO and LUMO energy levels. See Table 1 and the Supplementary Materials.

The HOMO and LUMO levels of **P1**, **P2**, and **P3** were obtained from the onsets of the oxidation and reduction potential [44], respectively, and are reported in Table 1 together with UV−vis spectroscopic data. The deviation between the optical and electrochemical results are in quite satisfactory agreement, as previously described in literature [45].

The energy levels of **P2** are similar to **P1**, consistently with the same conjugated backbone and lateral substituents. **P3** shows a variation of the HOMO and LUMO which is coherent to its polyfluorene backbone [39,40]. Importantly, as previously reported [46,47,48,49] the functionalization with the polar side chains does not interfere with the conjugated π-orbitals of the polyfluorene backbone and thus leads to no modification in the position of the HOMO and LUMO levels.

The different concentration in the copolymers of the sulfonated groups and alkyl chains is a tool to obtain different hydrophilic/hydrophobic characteristics: **P1** is the most hydrophilic owing to its two sulfonate groups per monomeric unit. The presence of only one polar side chain in **P2** and the presence of two long alkyl chains in **P3** are two approaches to reduce the CPEs hydrophilicity/polarity. This trend is confirmed by contact angle measurements on top of the **P1**, **P2**, and **P3** films using glycerol as a polar solvent. As shown in Figure S10 in the Supplementary Materials, the contact angles were found to be respectively 36° for **P1**, 49° for **P2**, and 68 ° for **P3**. Such reduction in the wettability to polar solvent going from **P1** to **P3** suggests that thanks to our design we were able to finely modulate the different hydrophilic/hydrophobic characteristics of the polymers and consequently the interaction with the a-polar active layer. 

As a consequence, **P1** has a good solubility in water but it is not soluble in pristine methanol or ethanol. **P2** shows an overall poor solubility in water/alcohol solvents but its dispersion in ethanol at low concentrations, 1 mg/mL, leads to stable suspensions which are suitable for film deposition by spin casting. **P3** can be also dissolved and deposited from ethanol at low concentrations (1 mg/mL). A good solubility in alcohol is particularly important with a view to preparing devices in an inverted configuration where a thin CPE film should be deposited on top of the hydrophobic active layer surface.

### 3.1. Self-Doping Behaviour

**Figure 2:**
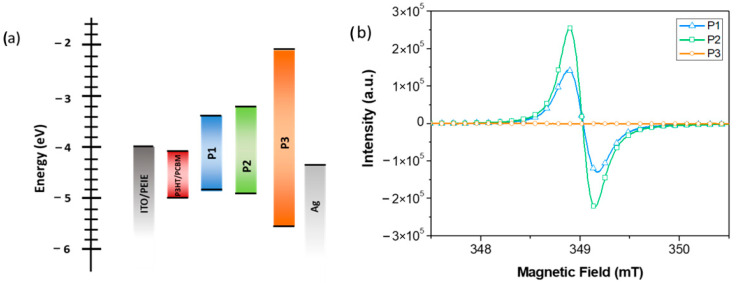
(**a**) Energy level of the materials used to fabricate inverted solar cells in this contribution; and (**b**) electron paramagnetic resonance (EPR) spectra of **P1**, **P2**, and **P3** in aqueous solution.

The self-doping behavior of **P1**, **P2**, and **P3** was investigated by electron paramagnetic resonance (EPR) measurements in aqueous solutions, see Figure 2b. A strong EPR symmetric signal consistent with the presence of unpaired electrons is observed for both **P1** and **P2**, with peak to peak linewidths and calculated g values respectively of 0.28 mT and 1.9955 for **P1** and 0.25 mT and 1.99314 for **P2**, which are the typical signatures of polaron formation in conjugated polymers [34,36]. It is important to underline that the values of the integrals are comparable and therefore the signal of the **P2** polymer is more intense than that of the **P1** polymer. Moreover, as it is well known, EPR is an intrinsically quantitative technique, since the EPR signals are due to the number of the excited spins [50]. As a consequence, at the same temperature and experimental conditions, EPR signal heights and, even more so double integrals, are comparable.

**Figure 3:**
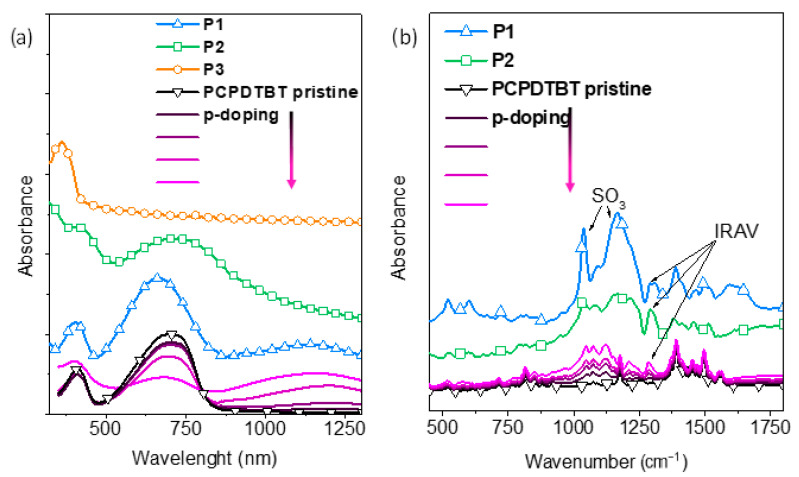
(**a**) UV/Vis-NIR absorption spectra of **P1**, **P2**, and **P3** films and (**b**) IR absorption spectra of **P2** and **P3** films with a pristine PCPDTBT film during its p-doping through I2 vapor exposure.

With regard to **P3**, no evidence of polaronic features is detected in the EPR spectrum. Polarons formation was previously reported for a **P1**-type CPE in aqueous solution and it was explained by a self-doping mechanism [33,34] in presence of a proton source like water [51]. Similarly, the polaronic features in the **P2** EPR spectrum indicate that self-doping in solution is occurring also when the PCPDTBT backbone is bearing only one alkylsulfonate lateral group per monomeric unit. On the other side, the absence of polarons in the **P3** EPR spectrum is consistent with the fact that self-doping can only take place in CPEs with a relatively low ionization potential, as is the case for **P1** and **P2**, but not for polyfluorene-based CPEs such as **P3** which have a high ionization potential/deep HOMO energy level. The UV/Vis-NIR absorption spectra of **P1**, **P2**, and **P3** films are reported in Figure 3a. The spectrum of **P1** exhibits two main bands centered at 405 nm and 660 nm that are peculiar for polymers with conjugated ciclopentadithiophene-benzothiadiazole (PCPDTBT) backbones and a weak and broad band peaked at 1140 nm which could be ascribed to the formation of polarons (radical cations) which are stabilized by the pendant sulfonate groups [34]. As shown in Figure 3a, a similar band is observed upon doping a pristine PCPDTBT film through exposure to I_2_ vapors. This confirms a polaronic transition assignment for the NIR absorption feature of the **P1** film absorption spectrum. The **P2** spectrum exhibits two main absorption bands, peaking respectively at 410 and 715 nm, which are similarly assigned to the PCPDTBT backbone transitions. The **P2** D-A (donor- acceptor) band at 715 nm has a broad tail in the NIR region which could arise from light scattering effects. A scattering tail is also observed in the absorption spectrum of a **P2** dilute solution, indicating that **P2** is partially aggregated even in diluted solution (0.1 mg/mL in ethanol or water, see Figure S2 in Supplementary Materials). The granular morphology in the solid state, evident from the atomic force microscopy (AFM) images reported in Figure S3, confirms the presence of aggregation and it is consistent to significant light scattering from **P2** films. As a matter of fact, the **P2** polarons spectral features, already evidenced by EPR spectra, are probably covered by the mentioned scattering, in the **P2** film absorption spectrum. Consequently, we can reasonably suppose that, similar to the well-known **P1**, self-doping is maintained also in **P2** films. The **P3** film shows a π-π* transition peak at 365 nm as expected for a polyfluorene backbone. No polaronic features are detected, which is consistent to the previously discussed absence of self-doping in solution for this fluorene-based CPE. 

The self-doping of CPEs films can be monitored by IR (Infra-Red) absorption spectroscopy. The absorption spectral signatures of polaronic charged states in conjugated polymers consist of both electronic transitions and IR vibrational modes, the so-called IRAV bands [52,53]. Figure 3b displays the IR absorption spectral pattern of **P1** and **P2**, with a pristine and I_2_-doped PCPDTBT for comparison. The **P1** and **P2** IR spectra show two intense and broad peaks at 1035 and 1165 cm^−1^. These bands are attributed to the symmetric and antisymmetric stretching modes of the SO_3_^−^ groups [54]. In **P3**, which has the same alkylsulfonate side chains, similar intense SO_3_^−^ stretching bands are observed (Figure S13, Supplementary Materials). Both **P1** and **P2** polymers show a weak band at 1285 cm^−1^, that is not observed in the **P3** spectrum of Figure S3. This suggests that an alkylsulfonate vibrational mode cannot account for this feature. Interestingly, this weak band does not match the pristine PCPDTBT IR spectrum but instead has a close similarity to one of the IRAV bands growing up upon p-doping PCPDTBT with I_2_ vapors. On the basis of these considerations, we suggest that the band observed at 1285 cm^−1^ in the IR spectra of **P1** and **P2** is an IRAV band of the conjugated backbone arising from polaronic charged defects. These features support our previous conjecture that self-doping is maintained also in the **P2** films. To sum up, both **P1** and **P2** display self-doping behavior, whereas **P3** does not. We will show in the following paragraphs that self-doping is one of the important characteristics of anionic CPEs for attaining effective interfacial anode modification in OSCs devices with inverted geometry. 

### 3.2. OSCs Devices Characterization 

Direct geometry device: We have characterized the copolymers’ interlayers in devices with direct geometry, made with a P3HT:PC_61_BM active layer, taking a standard PEDOT:PSS anode modifier as a reference. The devices architecture and PV characteristics are reported in the Supplementary Materials, Figure S14 and Table S2, while the devices’ assembly procedures are given in the experimental section. The **P1** devices exhibited the best PV performances with a power conversion efficiency (PCE) of 2.17, with comparable PV characteristics to the PEDOT:PSS reference device (see Table S1). A similar trend was already reported by the Bazan group using a **P1**-type CPE interlayer in P3HT:PC_61_BM devices [27]. Interestingly, the relatively high fill factor obtained using **P1** (0.58), brings support for an effective hole extraction when using **P1.** This can be ascribed to a combination of factors, including the good matching among the HOMO energy levels of **P1** and P3HT, which is preventing a barrier formation to hole extraction; and the self-doping of **P1**, which is known to induce beneficial effects for the hole transport and for the engineering of the ITO electrode work function [22]. With regard to **P2**, a reduction of the PV performances were observed (PCE 1.39%). **P2** has the same backbone/electronic features than **P1** but lower processability, owing to the previously mentioned low solubility and aggregates formation in solution, that affects its film forming properties as confirmed by the presence of thick aggregates with granular morphology in the AFM images (see Figure S3). Such features can account for the lower performances obtained with **P2** instead of **P1.** Using **P3** as anode interlayer, there is a drastic drop of the PV performances, similarly to the previously reported pristine anionic CPE AILs, with a high energy gap and no self-doping, as **P3** [23]. Kelvin probe measurements were used to investigate how the copolymers’ interlayers modify the ITO electrode work function (see Figure S15 in the Supplementary Materials). While the effective work function of the ITO electrode decreased from 4.8 eV to 4.7 eV with the **P3** interlayer, an increase to 5.02 eV and 4.98 eV was observed respectively with **P1** and **P2**, as expected for self-doped anionic CPEs [23]. Hence, the hole selectivity of the ITO electrode increases when using **P1** and **P2**, facilitating charge extraction, but it is reduced with **P3**. 

**Figure 4:**
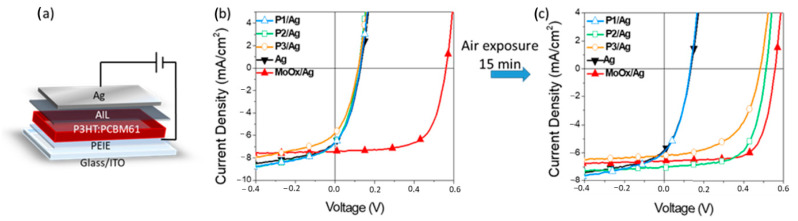
(**a**) Inverted devices architecture; (**b,c**) current density–voltage (J–V) curves under AM1.5 G irradiation at 100 mW/cm^2^. (**b**) before air exposure; and (**c**) after 15 min of air exposure.

Inverted geometry device: In order to investigate the key characteristics that anionic CPEs should have to obtain solution-processable anode interlayers for inverted OSC devices, we have tested **P1**, **P2**, and **P3** interlayers in P3HT:PC_61_BM-based devices, with a device architecture displayed in Figure 4. PEIE thin film was used as cathode interlayer on top of the ITO electrode. This non-conjugated amino-containing polymer interlayer affords good stability and efficient performance when applied in fullerene-based polymeric solar cells [55,56]. For this study, PEIE offers the advantage of being scarcely affected by the air soaking treatments herein reported. In inverted geometry the interlayers are deposited on top of the hydrophobic active-layer surface and to increase the wettability we used ethanol for the AILs processing; this was possible for **P2** and **P3**, but not for **P1** which needs a water/ethanol mixture to be dissolved and processed. The details of the devices’ assembly procedures are given in the experimental section. The PV characteristics are reported in Figure 4, Figure S16 in the Supplementary Materials, and Table 2.

The typical current density–voltage (J–V) curves of the devices featuring the polymeric interlayers **P1**, **P2**, and **P3** are depicted in Figure 4b; we have taken for comparison a device with a state-of-the-art evaporated MoOx interlayer (MoOx/Ag) and a device prepared without any AIL (Ag). The PV characteristics are summarized in Table 2. The devices with the copolymer interlayers showed poor performances, with PCEs around 0.3–0.25%, similar to the Ag devices and about one order of magnitude lower than the reference MoOx/Ag devices, with a PCE around 2.5%. In one of our previous works we have highlighted that the air exposure of devices with polar polymers interfacial layers improved their performances [57]. Interestingly, after a short air soaking treatment of 15 min, a significant enhancement of the PV performances was observed in the **P2** and **P3** devices, see Figure 4c and Table 2. Namely, the devices made with **P2** reached the best performances, comparable to the reference MoOx/Ag device, with a PCE of 2.62%; in **P3** devices, the PCE was 1.9%. In contrast to the **P2** and **P3** devices and irrespective to air exposure, **P1**-based devices showed the same poor performances of the Ag devices, prepared without any AIL. Such poor **P1** functionality in inverted devices might arise from the strong hydrophilic character of **P1**, leading to scarce adhesion and film formation on top of the hydrophobic P3HT:PC_61_BM active layer.

**Figure 5:**
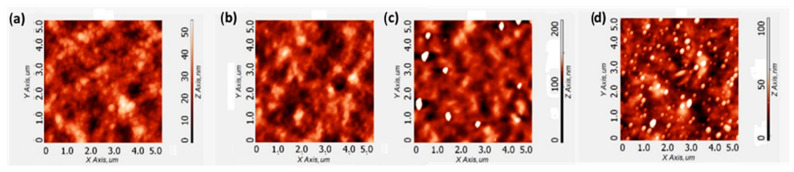
Surface topographic AFM images of (**a**) active layer; (**b**) active layer/**P1**; (**c**) active layer/**P2**; and (**d**) active layer/**P3** deposition. RMS values: 8.5 nm, 8.2 nm, 10.9 nm, 10.6 nm for (**a**–**d**), respectively. The active layer and CPE depositions were carried out in the devices assembly conditions.

The active layer (AL) coverage by the copolymer interlayers was analyzed by AFM. As shown in Figure 5, the surface morphology after **P1** deposition is similar to the AL substrate, thus suggesting a bad AL coverage by the **P1** interlayer. On the other hand, after **P2** and **P3** deposition, the morphology and roughness are significantly different. RMS values are reported in Figure 5 and are consistent with the presence of **P2** and **P3** on top of the active layer. 

To confirm the adhesion of our polar CPEs to the active layer, we measured the water contact angles prior to and after the interlayers deposition. As reported in the Supplementary Materials, Figure S12, the AL water contact angle reduces respectively from 107° to 96° and 102° after the **P2** and **P3** interlayers deposition. Such increase of hydrophilicity arises from the adhesion of the **P2** and **P3** polar polymers on top of the AL hydrophobic surface. Interestingly, by spin casting **P1** the water contact angle remained the same (at 107° as for the active layer) revealing a scarce/absent adhesion of **P1** on the active layer. This explains the identical PV characteristics observed for the **P1** device and the control Ag device. 

The above observations highlight an important requirement that an AIL CPE for solution-processed inverted solar cells has to satisfy: a correct balance between the hydrophilic and hydrophobic parts. This ensures a good adhesion to the active layers while maintaining solution processing from orthogonal solvents, which is a key for devices multi-stacking fabrication. 

As depicted in Table 2, the pristine Ag devices exhibit quite low V_OC_, poor FF, relatively high R_s_, and low R_p_. Such features are the typical fingerprints of the bad hole selectivity of a silver electrode. As a matter of fact, the Ag work function was reported at –4.3 eV, while the P3HT HOMO energy level was around −5 eV. As such, there is a bad electrode-organic band alignment, leading to a barrier to hole extraction.

The insertion of the **P2** or **P3** interlayers in the devices, combined with an air soaking treatment (15 min), induces a significant enhancement of the V_OC_ and FF parameters as compared to the pristine Ag. This testifies the ability of the **P2** and **P3** interlayers to improve the hole collecting character of the top Ag electrode. As depicted, the V_OC_ and FF parameters of the **P2** and **P3** devices are reaching values close to the ones obtained with the state-of-the-art MoOx interlayer. This behavior evidences an overall effective modification of the silver electrode-active layer band alignment by the insertion of these copolymers interlayers. However, **P2** appears to be a more effective anode modifier than **P3,** as self-doping occurs in **P2** but not in **P3**. Self-doping should favor the hole transport within the **P2** interlayer, leading to beneficial effects for hole extraction in the devices. Besides self-doping, **P2** exhibits a better energy level alignment to the P3HT AL component than **P3**, see Figure 2. This reduces the barrier to hole collection at the AL/CPE interface when using **P2** rather than **P3** [25]. 

The above comparison of **P2** and **P3** AILs clearly highlights that the choice of the conjugated backbone is a key in the development of effective AILs for inverted solar cells. To favor hole collection at the top silver electrode, the conjugated backbone should be designed to grant self-doping and provide a good band energy alignment at the AL–AIL interface.

Interestingly, in direct geometry, **P3** did not function as anode interlayer. In inverted geometry, even if **P3** is a less effective AIL than **P2**, due to the mentioned absence of self-doping and poor band alignment the **P3** interlayer is able to induce a good hole collecting character to the top Ag electrode. Since both **P3** and **P2** have alkylsulfonate side groups, but different conjugated backbones, we deduce that the sulfonate side groups are playing a role in the anode engineering of the inverted solar cells. 

It should be noticed that the ability of the sulfonate groups to impart a hole collection character to the top electrode could be not necessarily relate to this peculiar side group, but just to its inherent hydrophilicity. A hydrophilic material close to an Ag electrode upon air exposure may induce a shift from the vacuum of the Ag electrode work function via an oxidation mechanism [58]. 

**Figure 6:**
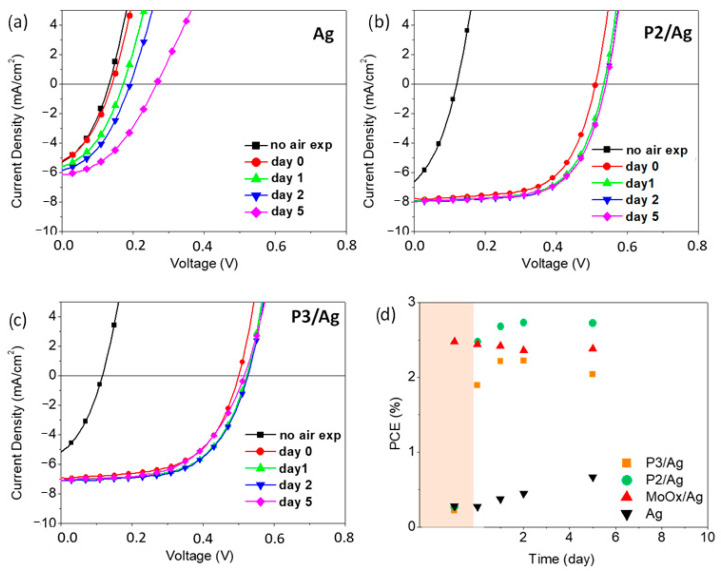
Evolution of the J–V curves and PCEs of devices with inverted geometry as a function of time of storage under ambient atmosphere under AM1.5G irradiation at 100 mW/cm^2^. Top electrode: (**a**) pristine Ag; (**b**) **P2**/Ag; (**c**) **P3**/Ag; and (**d**) PCEs of pristine Ag, MoOx/Ag, **P2**/Ag, and **P3**/Ag; before air exposure: pink background; after air exposure: white background. The data at day 0 refers to the devices exposed to air for 15 min, similar to Figure 4c.

**Figure 7:**
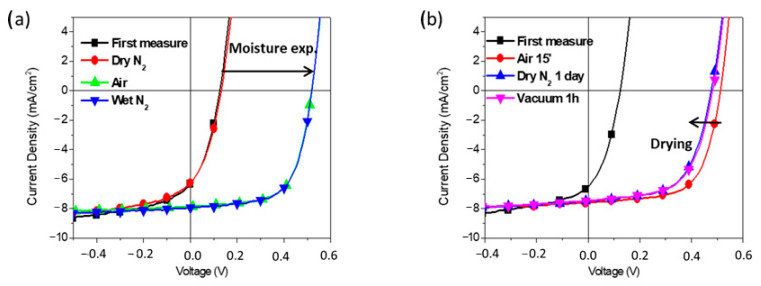
J–V curves of inverted devices stored for 15 min in different atmosphere conditions; (**a**) moisture exposure in air or in N_2_ atmosphere with a similar moisture content to air (50 % RH): Air and wet N_2_; first measure and dry N_2_ correspond respectively to a first and a second curve recorded after 15 min, for a device kept inside the glove box. (**b**) Drying of a device exposed to air for 15 min upon storage in a glovebox overnight prior and after 1 h under vacuum (10–7 atm.): Air 15 min, dry N_2_ overnight, 1 h vacuum; first measure is the J–V curve prior to air exposure.

In an attempt to clarify this issue, we have monitored, within a time scale of few days, the evolution of the J–V curves and PV characteristics of our devices versus their time of storage under air atmosphere. As depicted in Figure 6, with the **P2** and **P3** interlayers, most of the gain in hole selectivity of the top electrode is obtained rather quickly, at the very beginning of air exposure (“day 0” here corresponds to the previous 15 min air treatment). The Ag control device exhibits a completely different behavior upon aging under ambient atmosphere. At the very beginning of air exposure, the PV characteristics are not substantially affected. For longer times, a steady and continuous gain in hole selectivity of the top Ag electrode is observed. Such effect is ascribed to the formation of a thin oxide layer at the inner Ag surface, shifting the electrode work function to −5 eV [58]. It is known that this is a gradual process, driven by the slow diffusion of oxygen from the edges of the electrode [59]. For this reason, the gain in hole selectivity in the reference Ag devices is not completed in a few days [58,59]. By inserting the **P2** and **P3** interlayers, a faster process could be envisaged, owing to the hydrophilic nature of the CPEs which is attracting moisture at the buried silver. Moisture, in fact, provides a medium for adsorption of gases and the subsequent formation of an oxide layer [60,61]. However, a gradual oxidation process would be anyhow expected [18], which is by far different from the **P2** and **P3** trend depicted in Figure 6. For this reason, the formation of an Ag_2_O layer is not enough to explain the functional behavior of the **P2** and **P3** anode interlayers. This was indeed confirmed by monitoring the **P2** device PV performances after 15 min storage under different ambient conditions, see the J–V curves in Figure 7 and corresponding PV parameters in the Supplementary Materials. We have found that a N_2_ atmosphere, without oxygen but with a moisture content as in ambient air (50 % RH), is identically effective for the PV performances as a standard air soaking treatment. Therefore, it is moisture rather than oxygen which plays a role in the **P2** and **P3** functional behavior. As shown in Figure 7b, by exposing the devices back to dry N_2_ atmosphere and/or by drying them up by vacuum treatments, the anode modification is very stable and almost no reversibility is observed.

To summarize, the above results indicate that the mechanism that explains the common ability of **P2** and **P3** in modifying the top anode electrode is related to the combined effect of the sulfonate side groups and water molecules. Note that in fact water is a proton source able to dope this class of polymers, as recently reported by Bazan and coworkers [51].

The importance of sulfonate groups in the engineering of the top Ag electrode was also confirmed by using a PTB7-Th-based active layer (see the Supplementary Materials). Here, the non-optimal alignment of the levels between the PTB7-Th and AIL led to PV poor performance, but similarly to P3HT-based devices, a quite relevant gain in hole selectivity was also observed.

According to the above discussion, it has been identified that the choice of the polar group is another extremely important factor in the design of the AILs. We infer that the sulfonate moieties not only assure the solubility in polar orthogonal solvents, inducing self-doping via a right choice of the conjugated backbone, but also play a role for the anode engineering in inverted solar cells.

**Figure 8:**
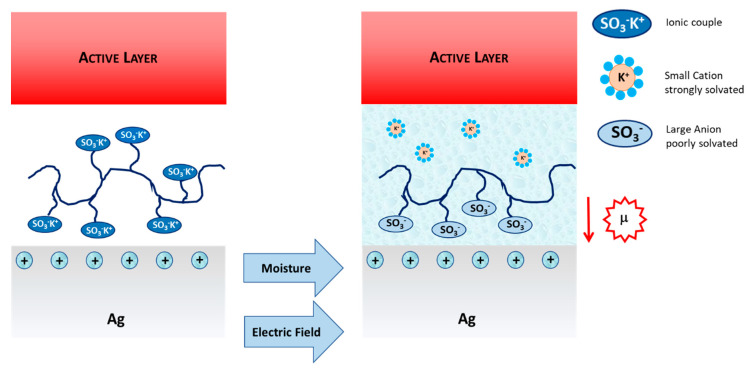
Proposed mechanism for moisture annealing.

However, a simple picture that may possibly explain this mechanism is proposed in the following Figure 8. In the presence of moisture/water, SO_3_^−^ anions get partially solvated and therefore the ionic pair with the alkaline metal K^+^ is weakened, gaining the freedom to better interact with the silver surface. Moisture may also facilitate the orientation of the sulfonate groups towards the metal interface by sweeping the voltage (electric field) in the device. As a result, dipoles oriented towards the inner silver electrode should be formed, shifting from vacuum the silver electrode work function. As a result, the hole collecting character of the top electrode is improved.

## 4. Conclusions

In this work we have conducted a study about the unexplored application of sulfonate anionic CPE solution-processable anode interlayer materials in inverted organic photovoltaics. Based on the results obtained in our study it is possible to establish a good understanding of AIL structure–property–PV performance relationships for inverted devices applications. In fact, by designing and investigating the functional behavior of three different polymers **P1**, **P2**, and **P3** bearing a different number of sulfonate groups and modifications of their conjugated backbone, we could assess the important material features that should be taken in account for the development of effective AIL CPE materials for inverted OSC devices. First, it is mandatory to develop anionic CPEs with good wettability to the active layer and this can be achieved by the correct balancing of hydrophilic and hydrophobic substituents. Second, similarly to conventional direct geometry devices, the conjugate backbone should be suitably designed to ensure the self-doping of the CPE materials and grant a good energy level match with the photoactive layer. Finally, the choice of the polar groups is another important factor in the design of the AILs. The sulfonate moieties not only assure the solubility in polar orthogonal solvents, induce self-doping via a right choice of the conjugated backbone, but also play, combined with moisture exposure, a role for the anode engineering in inverted solar cells. Moisture annealing is a simple, easily accessible, and low cost procedure and could be a valuable alternative to electrical or thermal annealing. However a more comprehensive understanding of the importance of sulfonate for the self-doping mechanism of CPEs and its effect on charge transport and mobility is required to improve the use and design of this class of polymers. We believe that our insight could give a valuable contribution to the advancement in the development of engineering all polymeric solution-processable inverted solar cells.

## Figures and Tables

**Table 1:** Optical properties and HOMO and LUMO energy levels of **P1**, **P2**, and **P3**.

Polymer	λ_max_ (nm)	λ_onset_ (nm)	E_g_^a^ (eV)	HOMO (eV)	LUMO (eV)	E_g_^b^ (eV)
**P1**	630	898	1.38	−4.87	−3.31	1.56
**P2**	719	947	1.31	−4.83	−3.13	1.50
**P3**	389	426	2.91	−5.50	−2.2	3.23

^a^ Estimated from the onset wavelength of the optical absorption in the solid state film (Figure 3a). ^b^ Calculated from the HOMO and LUMO level.

**Table 2:** Summary of the photovoltaic parameters ^a^ using pristine Ag or MoOx, **P1**, **P2**, and **P3** AILs before and after an air exposure treatment of 15 min.

Device	V_oc_ (V) ^a^	FF ^a^	J_sc_ (mA/cm²) ^a^	PCE (%) ^a^	R_s_^b^ (Ωcm^2^)	R_sh_^c^ (kΩcm^2^)
Before air exposure treatment						
**Ag**	0.13	0.365	6.91	0.33 ± 0.01	9.19	1.70
**MoOx**	0.56	0.645	6.86	2.48 ± 0.2	6.85	123.0
**P1**	0.13	0.356	6.67	0.31 ± 0.01	9.17	2.26
**P2**	0.12	0.355	6.30	0.28 ± 0.01	8.21	1.37
**P3**	0.12	0.349	6.20	0.26 ± 0.01	9.99	0.79
After air exposure treatment (15 min)						
**Ag**	0.13	0.382	6.54	0.33 ± 0.01	9.86	1.71
**MoOx**	0.56	0.693	6.83	2.63 ± 0.12	6.87	174.9
**P1**	0.13	0.382	6.64	0.33 ± 0.01	9.94	3.49
**P2**	0.51	0.653	7.86	2.62 ± 0.15	7.20	31.03
**P3**	0.48	0.527	7.54	1.90 ± 0.2	9.42	64.46

^a^ average values across 12 devices; ^b^ R_s_ are calculated from the light J–V curve inverse slope at voltages around the V_OC_; ^c^ R_sh_ are calculated from the dark J–V curves at voltages around V_OC_ = 0.

## Data Availability

Data is contained within the article and supplementary material.

## Supplementary Materials

The following are available online, H-NMR spectra, GPC spectra, IR and UV-Vis absorption spectra, AFM images, contact angle, Kelvin probe and photovoltaic characterizations.
