# Supplementary material for: Sulfonate-Conjugated Polyelectrolytes as Anode Interfacial Layers in Inverted Organic Solar Cells
Source: Molecules. 2021 Feb 2;26(3):763. doi: 10.3390/molecules26030763 (PMC7867262; doi:10.3390/molecules26030763)
Supplement: Supplementary file 1 [file molecules-26-00763-s001.pdf]

# Electronics Supplementary Information (ESI)

# Solution-Processed Conjugated Polyelectrolytes as Anode Interfacial Layers in Inverted Organic Solar Cells

**Elisa Lassi,[1]‡ Benedetta M. Squeo[1]‡, Roberto Sorrentino[1], Guido Scavia[1], Simona Mrakic-Sposta[2], Maristella Gussoni[1], Barbara Vercelli[3], Francesco Galeotti[1], Mariacecilia Pasini[1]* and Silvia Luzzati[1]***

[1] Istituto di Scienze e Tecnologie Chimiche "G. Natta" - SCITEC, Consiglio Nazionale delle Ricer-che, CNR-SCITEC , via Corti 12, 20133 Milan, Italy;

[2] Istituto di Bioimmagini e Fisiologia Molecolare, Consiglio Nazionale delle Ricerche, CNR-IBFM, Via Fratelli Cervi, 93, 20090 Segrate, Italy

[3]. Istituto di Chimica della Materia Condensata e di Tecnologie per l'Energia, Consiglio Nazionale delle Ricerche, CNR-ICMATE, Via Roberto Cozzi, 53 - 20125 Milan, Italy

‡ E. Lassi and B.M. Squeo contributed equally to this work.

* Correspondence: M.P. : mariacecilia.pasini@scitec.cnr.it; S.L.: silvia.luzzati@scitec.cnr.it

## Experimental

The UV−Vis absorption spectra were obtained with a Perkin Elmer Lambda 900 spectrometer. IR spectra have been recorded with a Bruker IFS66 FTIR. AFM and Kelvin probe measurements have been performed with a commercial AFM KPFM NTMDT NTEGRA, with noncontact/tapping cantilevers (NSG10) for height images and with conductive Pt/Ir coated cantilevers (NSG10/Pt) for surface potential images. The kelvin measurements have been performed under a controlled $N_2$ atmosphere. The thickness of the layers was measured by a profilometer (KLA Tencor, P-6). Contact angle measurements were performed with an OCA20 instrument (Dataphysics Co., Germany), equipped with a photo camera CCD and a 500 mL Hamilton syringe to dispense liquid droplets. EPR experiments were carried out by means of an e-scan EPR spectrometer (Bruker, Germany) operating at the common X-band microwave frequency (~9.8 GHz). The instrument was interfaced to a temperature and gas controller Bio III unit (Noxigen Science Transfer & Diagnostics GmbH, Germany). $^1$H-NMR spectra were recorded with a Bruker DRX 600 MHz spectrometer and Bruker ARX 400MHz spectrometer. Gel permeation chromatography (GPC) measurements were carried out on a Waters SEC (Size Exclusion Chromatography) system consisting of a pump, a differential refractometer, and light scattering spectrometer as the detector. The columns were two PL Polargel (M+L) columns; solvents used were DMSO and LiBr 0.1M with a solvent flow rate of 0.6 ml/min at 80°C; the calibration curve was constructed with narrow MWD PMMA standard .

## Electrochemical Measurements

Electrochemistry was performed at room temperature in acetonitrile under nitrogen in three electrode cells. The counter electrode was platinum; reference electrode was $Ag/Ag^+$ (0.1 m $AgNO_3$ in acetonitrile[1], 0.34 V vs SCE, −4.73 V vs vacuum); supporting electrolyte was 0.1 m tetrabutylammonium perchlorate (TBAP). **P1**, **P2**, and **P3** films were casted onto carbon glass electrodes at 80–90 °C from solutions in water, ethanol/water, and ethanol, respectively. The reference electrode was calibrated vs SCE (saturated calomel electrode). The voltammetric apparatus used was the Metrohm Autolab 128N potentiostat/galvanostat. The working electrode for cyclic voltammetry (CV) was a glassy-carbon (CG) minidisc electrode (0.2 cm$^2$), with a scan speed of 100 mVs$^{-1}$. HOMO levels were estimated according to the equation $E_{HOMO} = -(Eox + 4.39 + 0.34)$.[2]

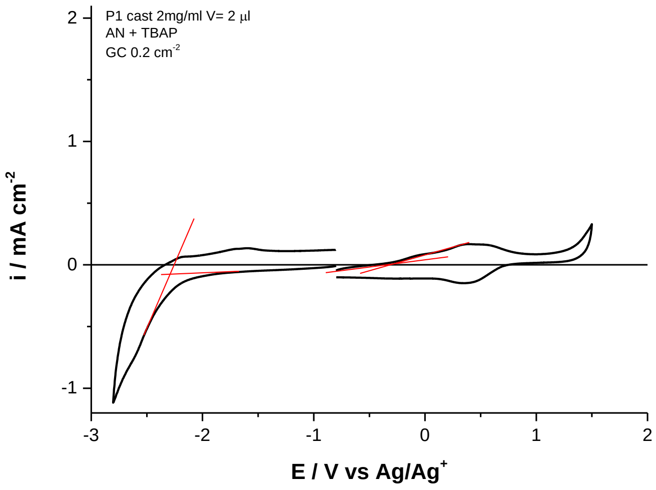


**Figure S0.** Cyclic voltammetry plot of **P1**

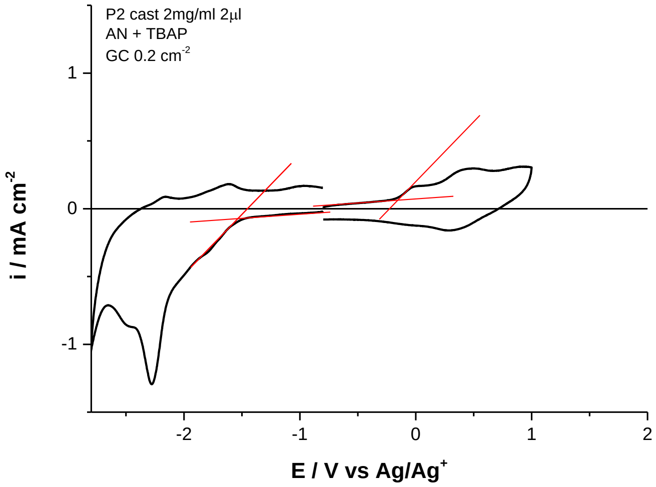


**Figure S1.** Cyclic voltammetry plot of **P2.**

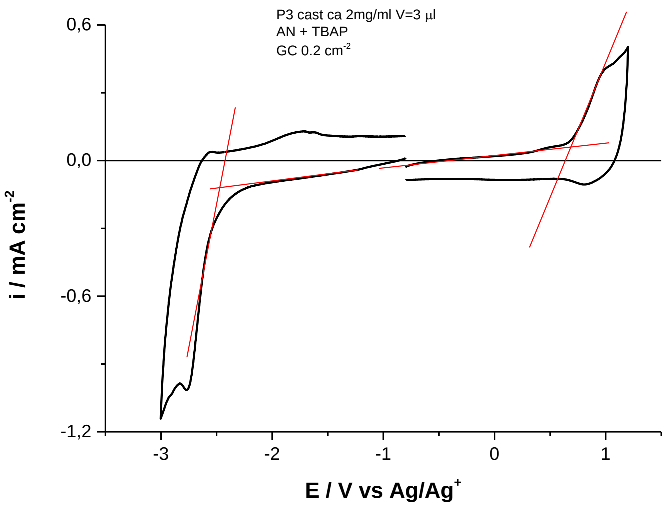


**Figure S2.** Cyclic voltammetry plot of **P3.**

## EPR Measurements

EPR experiments were carried out by means of an e-scan EPR spectrometer (Bruker, Germany) operating at the common X-band microwave frequency (~9.8 GHz). The instrument was interfaced to a temperature and gas controller Bio III unit (Noxigen Science Transfer & Diagnostics GmbH, Germany). All the spectra were recorded at 25 °C, adopting the same acquisition parameters: 10 scans on average, 5.24 s sweep time, 2.24 G modulation amplitude, 86 kHz modulation frequency, 21.9 mW microwave power, at a frequency of 9.786 GHz. DPPH was used as a standard reference sample (Hutchison and Pastor 1951; Feher and Kip 1955) for g-value determination. Spectra were analyzed by using a standard software (Win EPR version 2.11).

## P1, P2, and P3 Interlayer Thicknesses

**P1** interlayer thicknesses were measured through AFM and profilometer to be around 10–12 nm when deposited on ITO.

**P2** films are brittle, so that the profilometer scratched the film during the measurement both for deposition on top of the ITO and on the active layer. To obtain some information about the film thickness we repeated the profilometer sweeps several times on the same position, each time taking away a part of the **P2** film. As a matter of fact, after 5–10 sweeps, the step heights disappeared on the ITO while they reached a constant value, corresponding to the active layer thickness for inverted geometry, where **P2** was deposited on top of the active layer. The **P2** film thickness was evaluated from the step height difference among the first and last sweep. After measuring with this approach the **P2** film thicknesses at six different points of the substrates, we evaluated the **P2** average film thickness to be about 30 nm for the interlayers deposited on top of ITO, for the devices with direct geometry, and about 40 nm for the interlayers deposited on top of the active layer, for devices with inverted geometry.

**P3** film thicknesses are lower than 10 nm. Such films are too thin to be measured with AFM/profilometer techniques. In this case, the film thickness was evaluated from the intensity of the **P3** absorption peak, measured on a film deposited on a quartz substrate, used as an absorption coefficient for polyfluorene-based polar polymers previously extracted[3]. **P3** film thicknesses were evaluated to be around 5 nm.

## Schemes, Figures, and Tables

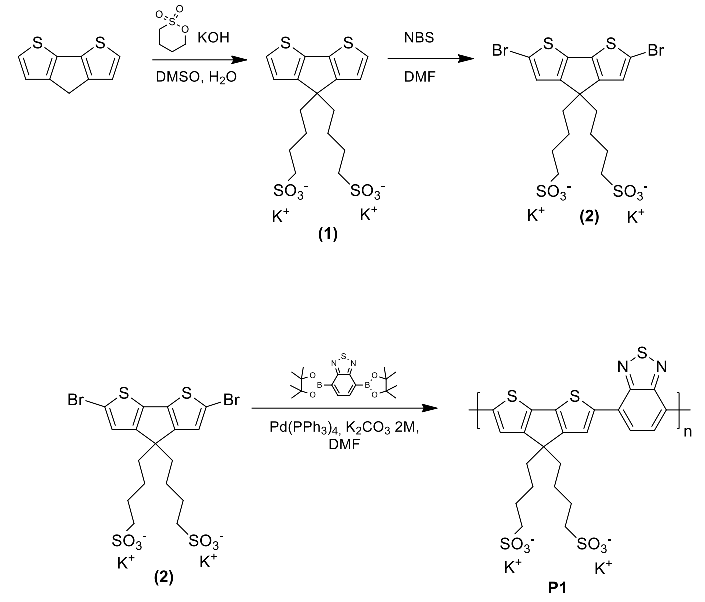


**Scheme 1.** Synthesis of **P1**.

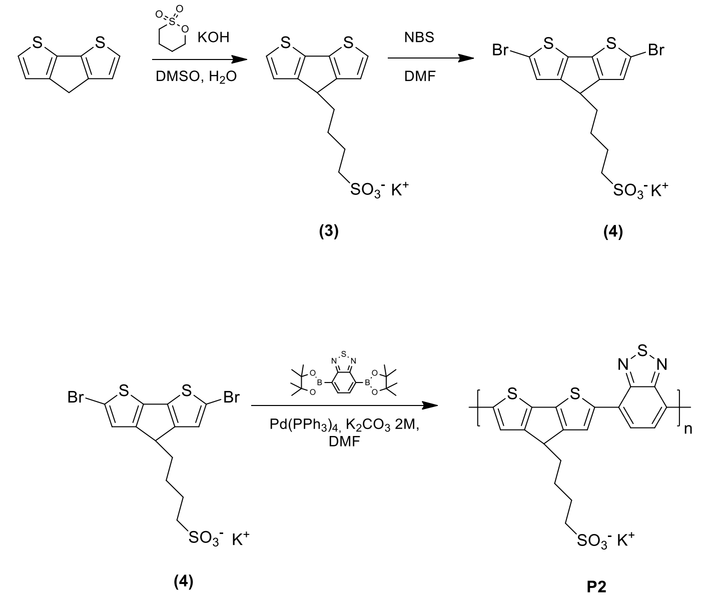


**Scheme 2.** Synthesis of **P2**.

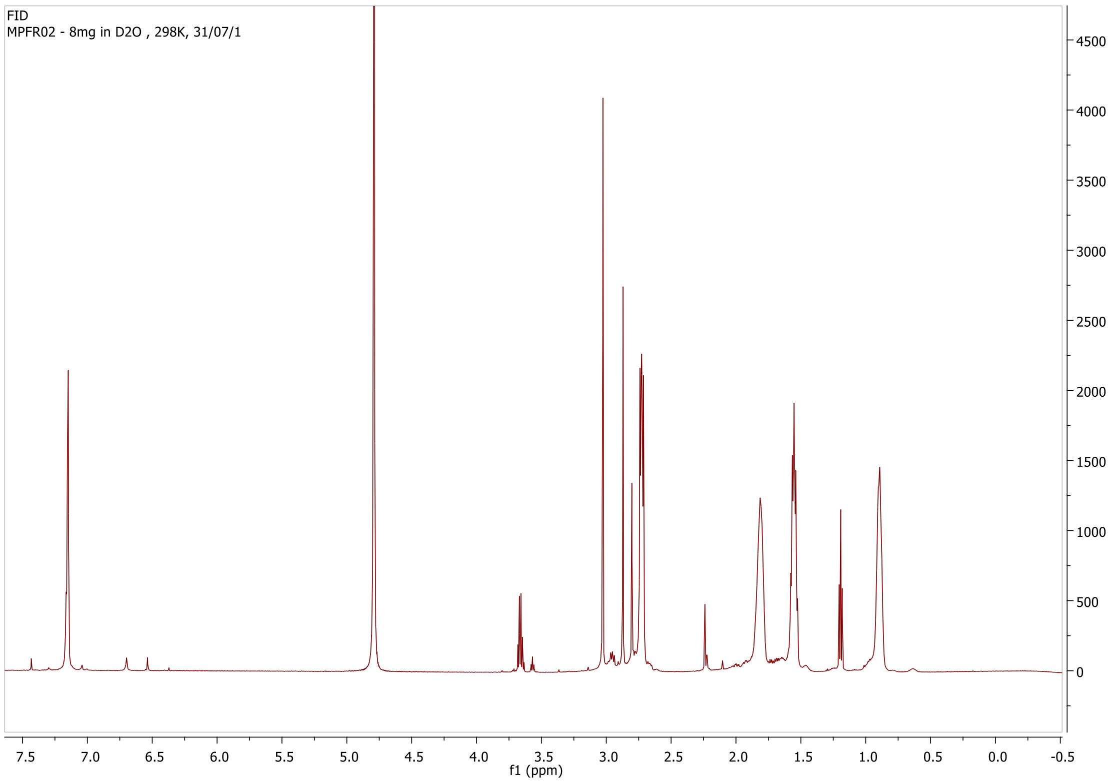


**Figure S3.** $^{1}H$ NMR spectrum of compound (**2**) in $D_2O$.The purity of the monomer is estimated to be around 95%.

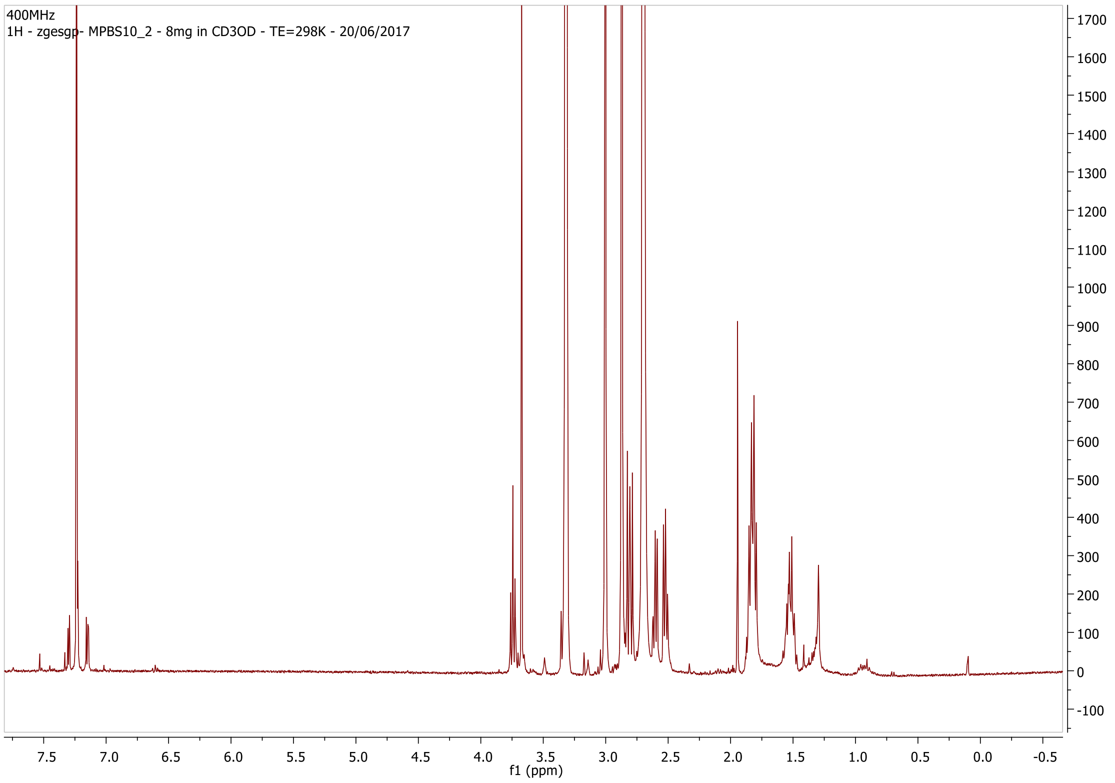


**Figure S4.** $^{1}H$-NMR spectrum of compound (**4**) in $CD_3OD$. The purity of the monomer is estimated to be around 94%.

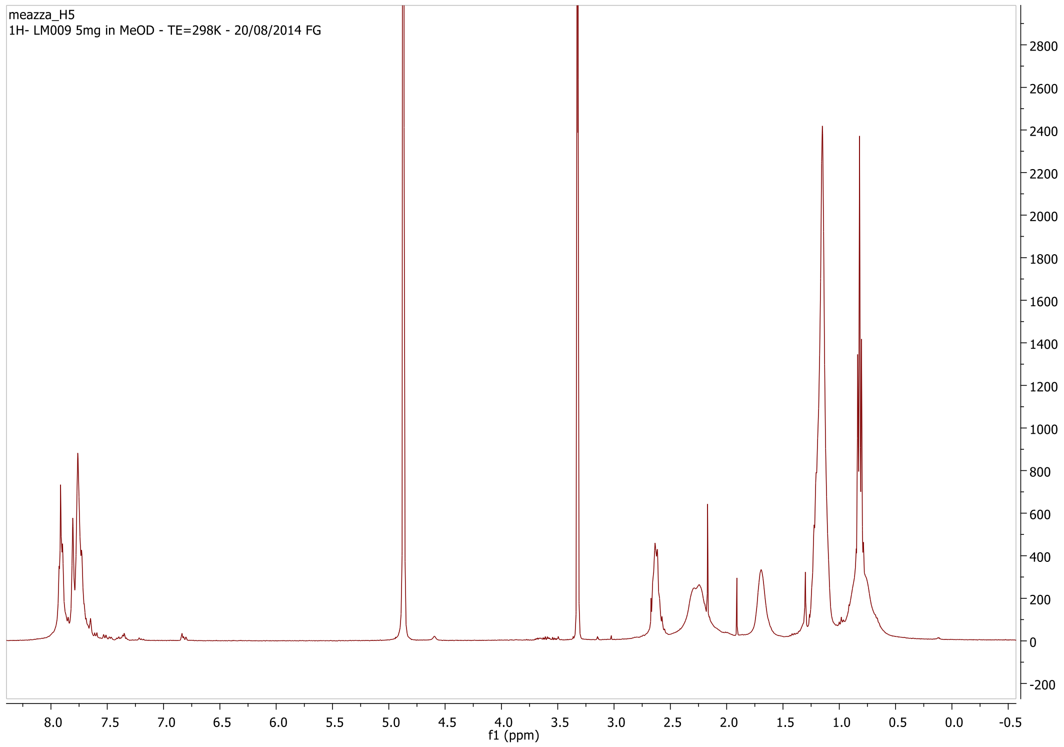


**Figure S5.** $^{1}$H NMR spectrum of compound **P3** in $CD_3OD$.

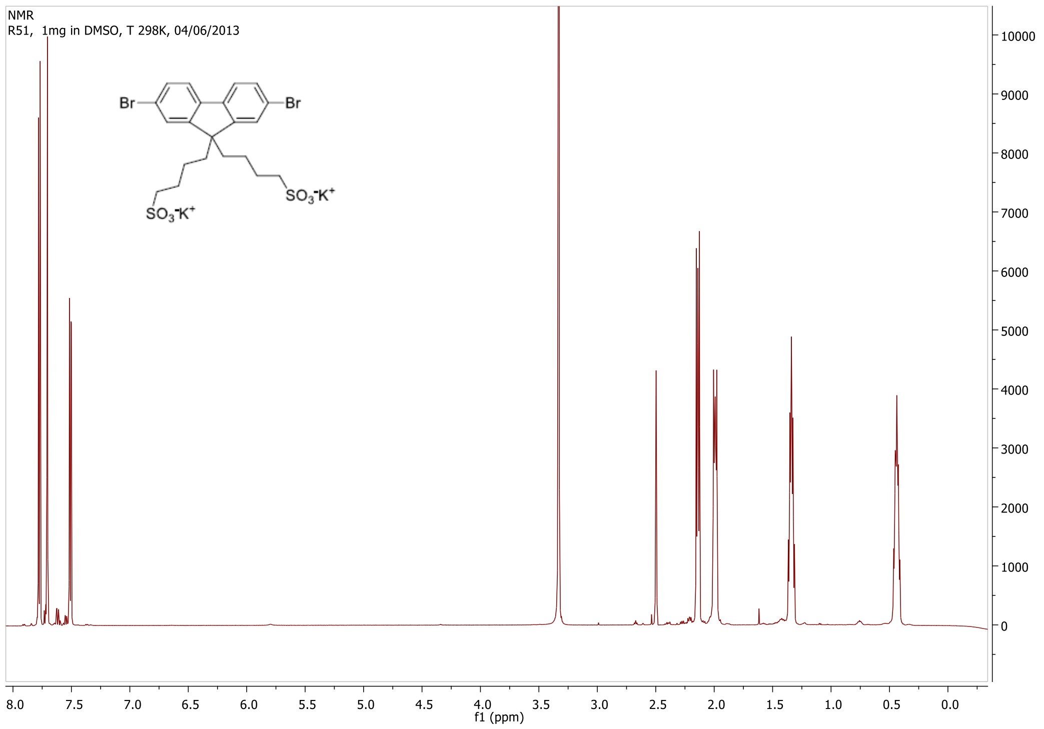


**Figure S6.** $^{1}$H-NMR spectrum of 2,7-dibromo-9,9-bis(4′-sulfonatobutyl)fluorene dipotassium. The purity of the monomer is estimated to be around 97%.

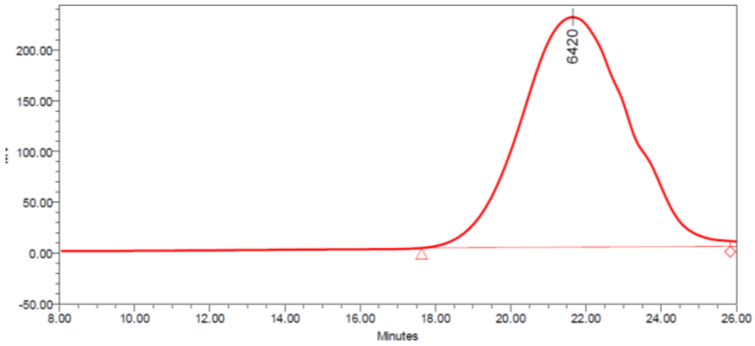


**Figure S7.** SEC spectrum of polymer **P1**.

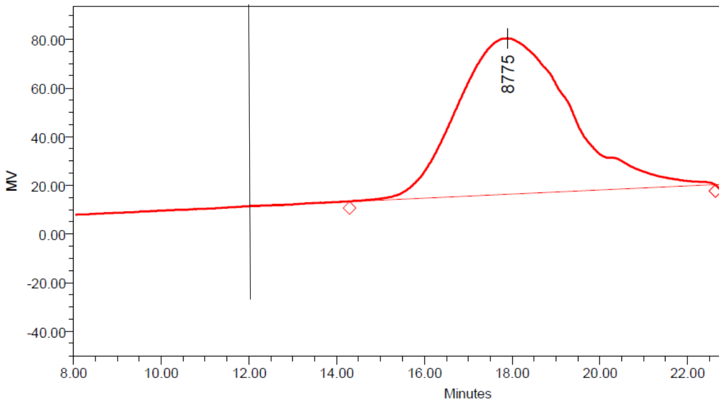


**Figure S8.** SEC spectrum of polymer **P2**.

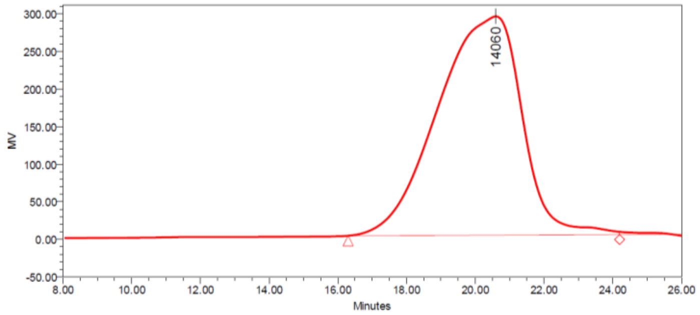


**Figure S9.** SEC spectrum of polymer **P3**.

**Table 1.** Molecular weight estimated with SEC.

| Polymer | Mn [Da] | Mw [Da] | Mw/Mn |
|---|---|---|---|
| **P1** | 3916 | 9246 | 2.36 |
| **P2** | 3194 | 10939 | 3.42 |
| **P3** | 14775 | 30912 | 2.09 |

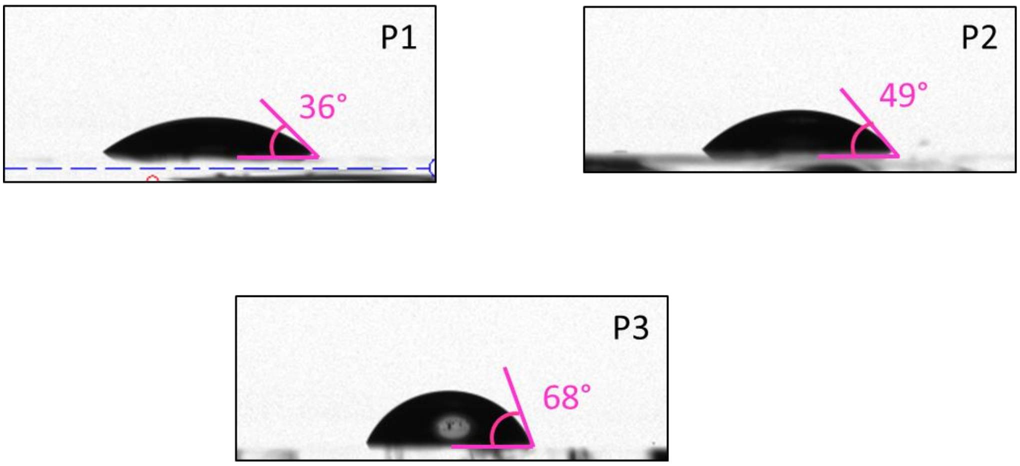


**Figure S10.** Contact angle with glycerol on **P1** (36 ± 0.8), **P2** (49 ± 1.1), and **P3** (68 ± 2.05) films deposited on glass treated with plasma for 10 min.

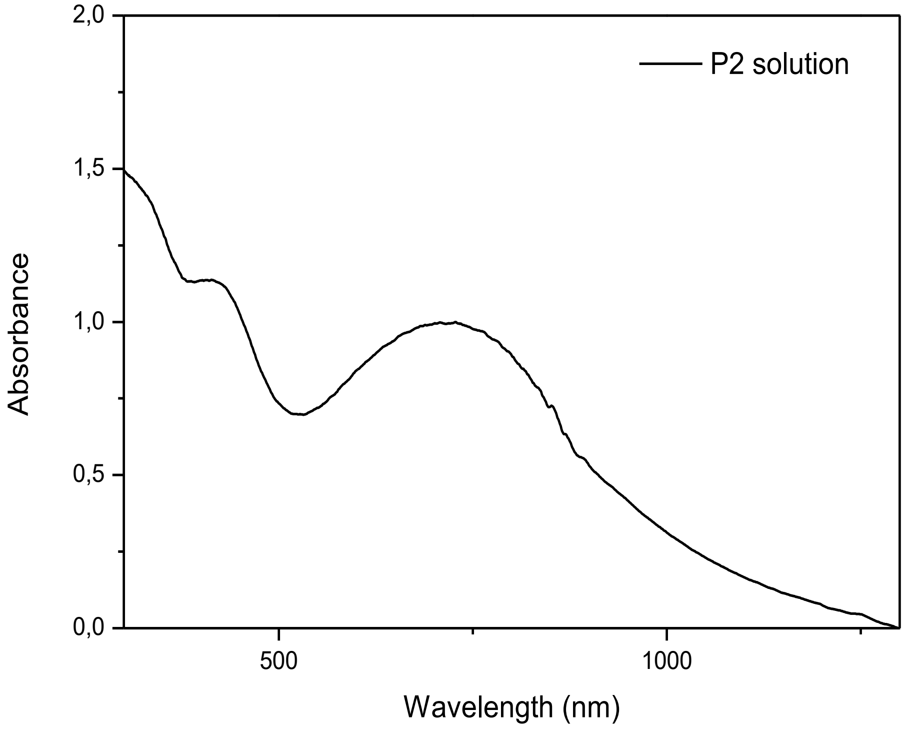


**Figure S11.** Absorption spectrum of **P2** solution in ethanol (0.1 mg/mL).

## AFM Images of P1, P2, and P3 Interlayers on ITO Substrates

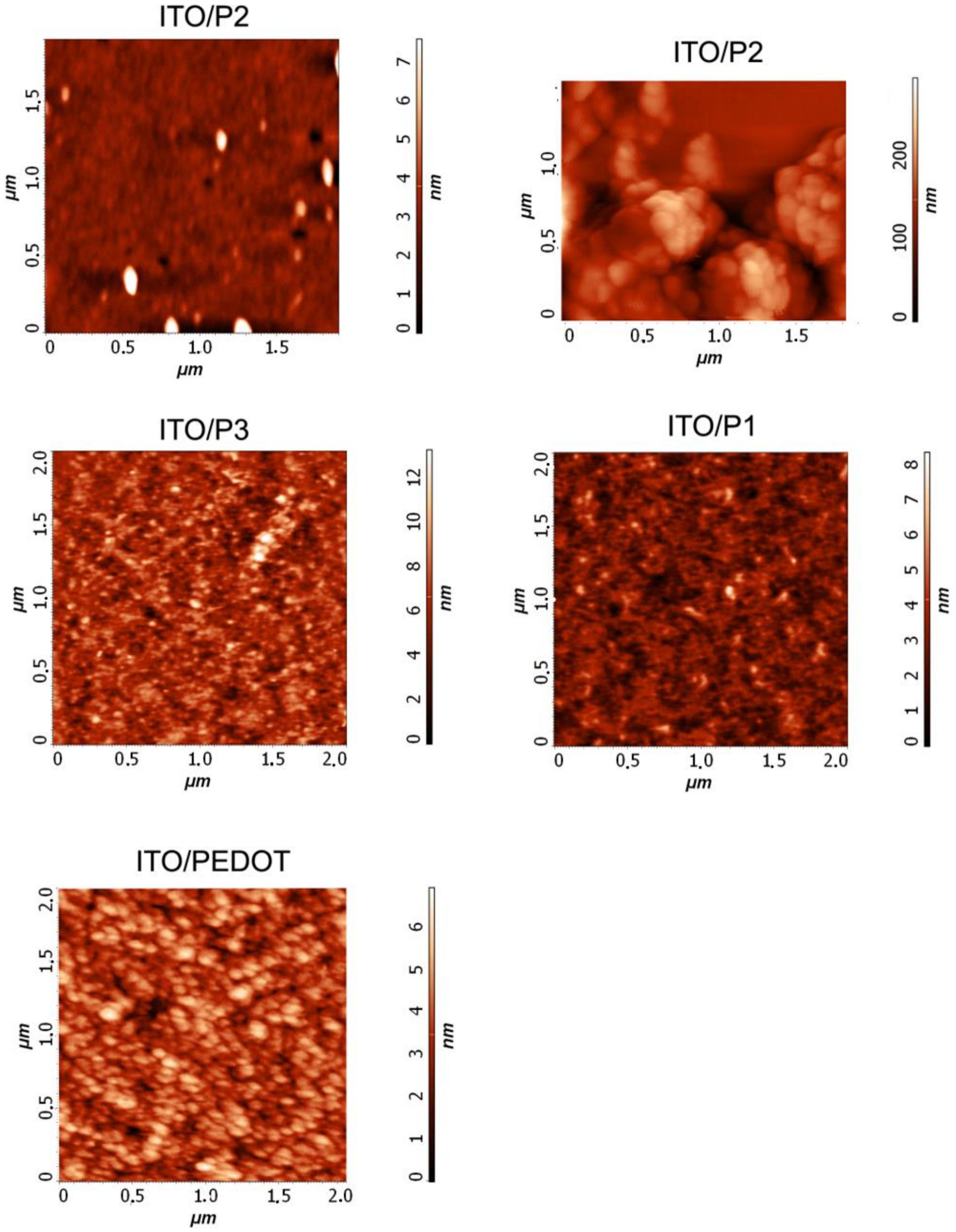


**Figure S12.** AFM images of **P1**, **P2**, **P3** and PEDOT:PSS films deposited on top of ITO.

**P1**, **P3** and PEDOT:PSS films exhibit an homogeneous surface on top of ITO. **P2** does not form a homogeneous film on the ITO substrates. In some parts, **P2** covers the ITO surface with a smooth film, similar to other interlayers, see first AFM image on the left. In other parts, the surface reveals the presence of extended and thick aggregates, with a granular morphology, see first AFM image on the right. Such features are likely arising from the aggregates in solution that are leading to important light scattering effect both in solution and in the films.

## IR Spectra

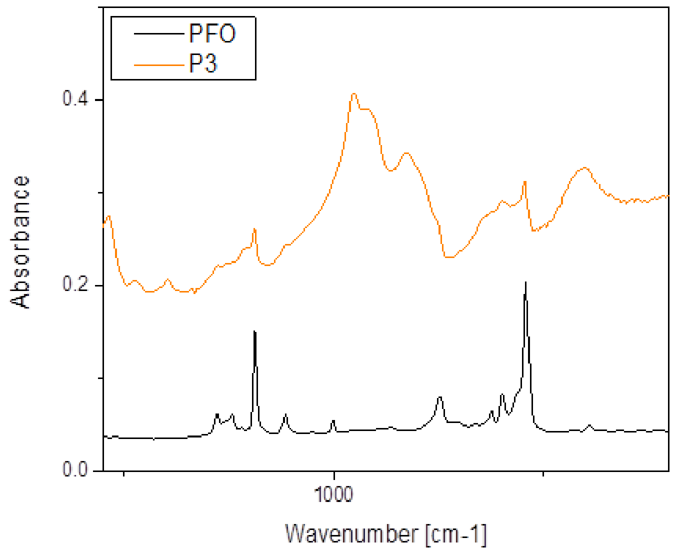


**Figure S13.** IR absorption spectra of polymer films, **P3** (orange line) compared with the PF backbone with alkylsubstituents only (black line).

## Direct Geometry Devices

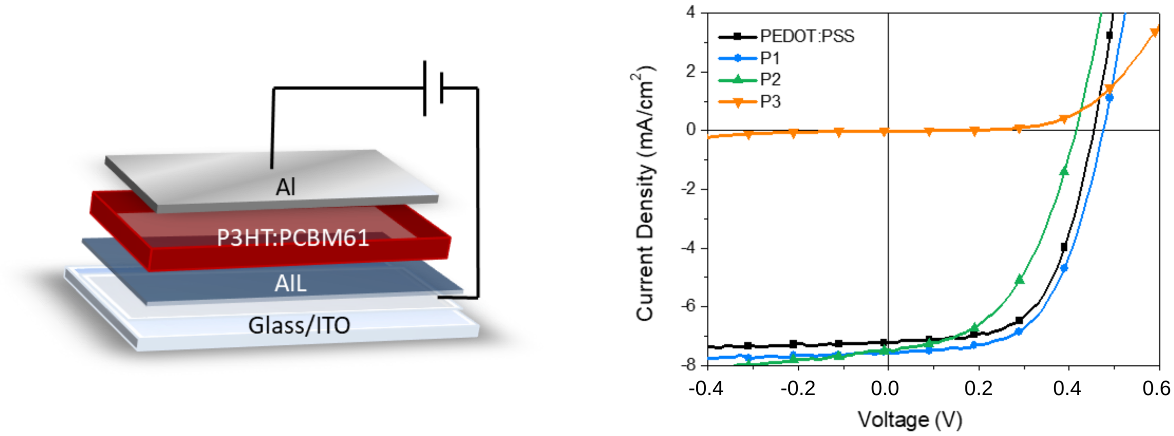


**Figure S14.** (**a**) Device structure used for conventional geometry P3HT:$PC_{61}BM$ devices and (**b**) J–V curves obtained using **P1** (blue), **P2** (green), and **P3** (orange) as AIL compared with PEDOT:PSS (black).

**Table S2.** Summary of the photovoltaic parameters using PEDOT:PSS, **P1**, **P2**, and **P3** as AIL in conventional geometry devices, average value over six devices.

| Interlayer (thickness) | $V_{oc}$ (V) | FF | $J_{sc}$ (mA/cm$^2$) | η (%) |
|---|---|---|---|---|
| **PEDOT:PSS** (35 nm) | 0.45 ± 0.01 | 0.59 ± 0.009 | 6.90 ± 0.27 | 1.84 ± 0.06 |
| **P1** (10 nm) | 0.48 ± 0.01 | 0.576 ± 0.014 | 7.86 ± 0.24 | 2.17 ± 0.14 |
| **P2** ( ~30 nm) | 0.41 ± 0.33 | 0.476 ± 0.015 | 7.13 ± 0.36 | 1.39 ± 0.12 |
| **P3** (5 nm) | 0.13 ± 0.06 | 0.268 ± 0.021 | 0.04 ± 0.021 | 0.001 |

**Kelvin Probe Measurements**

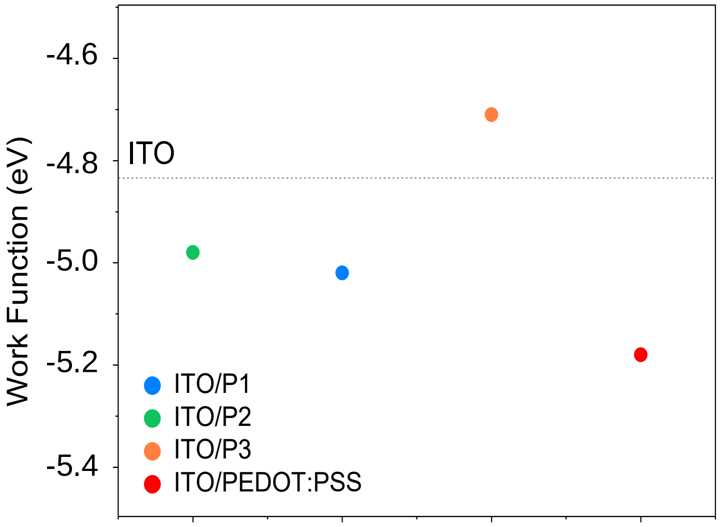


**Figure S15.** Effective work function of ITO/**P2** (green dot), ITO/**P1** (blue dot), ITO/**P3** (orange dot), and ITO/PEDOT:PSS (red dot) interlayers as measured via Kelvin probe force microscopy. The black dotted line corresponds to bare ITO (black dot line).

## EQE Spectra (Direct and Inverted Geometry)

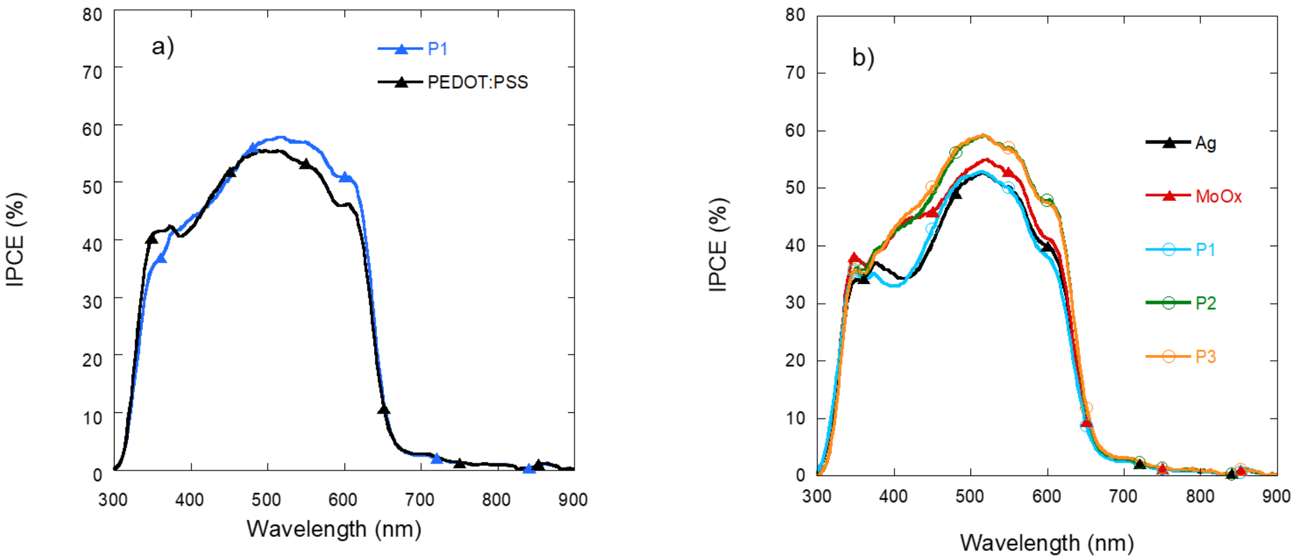


**Figure S16.** EQE spectra of P3HT:$PC_{61}BM$ devices with the AILs under study in **a**) direct geometry (J–V curves in Figure S14); **b**) inverted geometry ( J–V curves in Figure 4). The $J_{scEQE}$ (mA/cm$^2$), calculated from the EQE spectra ,are respectively: a) PEDOT:PSS (8.18), **P1** (8.55); and b) pristine Ag (7.18), MoOx (7.70), **P1** (7.09), **P2** (8.36), and **P3** (8.41).

## Contact angle measurements

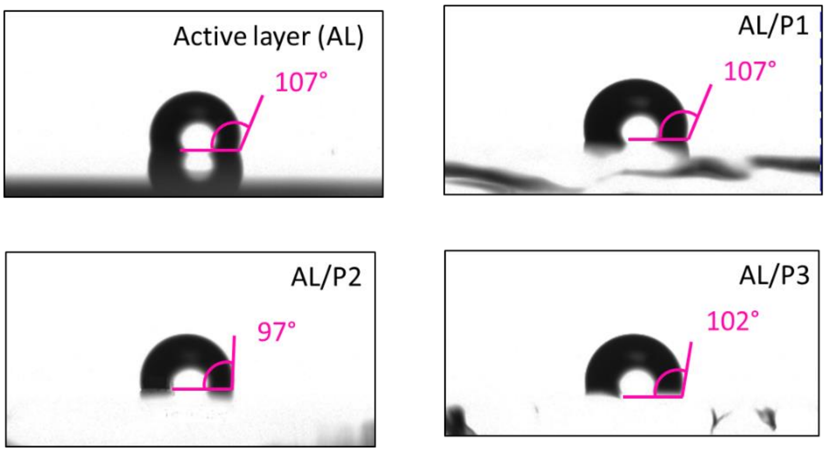


**Figure S17.** Contact angle with water on active layer (AL: 107 ± 1.3) and on the active layer with **P1** (107 ± 2.6), **P2** (97 ± 2.7), and **P3** (102 ± 1.26) as interlayers.

## PV Characteristics Upon Different Ambient Atmosphere Exposure Treatments

**Table S3.** PV characteristics of **P2** devices upon different ambient atmosphere exposure treatments, corresponding to the J–V curves of Figure 7.Exposure treatments after a first measure inside the glove box.

| 15 min Ambient Treatments | Voc (V) | FF (--) | Jsc (mA/cm²) | PCE (%) | Rs[a] (Ωcm²) |
|---|---|---|---|---|---|
| First measure* | 0.13 | 0.375 | 6.80 | 0.33 | 10.05 |
| AIR | 0.51 | 0.639 | 7.61 | 2.48 | 7.2 |
| $N_2$+ 50% RH | 0.51 | 0.614 | 7.59 | 2.13 | 7.32 |
| $N_2$ dry, in GB | 0.13 | 0.396 | 5.96 | 0.31 | 10.58 |

**Reversibility: Drying Treatments After 15 min Air Exposure.**

| Treatments | Voc (V) | FF (--) | Jsc (mA/cm²) | PCE (%) | Rs[a] (Ωcm²) |
|---|---|---|---|---|---|
| First measure* | 0.12 | 0.374 | 6.25 | 0.28 | 9.16 |
| 15 min air exposure | 0.51 | 0.634 | 8.20 | 2.65 | 8.01 |
| 24 h storage in dry $N_2$, GB | 0.47 | 0.603 | 7.24 | 2.05 | 10.26 |
| 24 h storage in dry $N_2$, GB + 1 h vacuum | 0.47 | 0.583 | 7.42 | 2.03 | 10.35 |

*first measure inside the glove box; [a] calculated from the J–V slope near the Voc.

## PTB7-Th:$PC_{71}BM$ Devices with Inverted Geometry

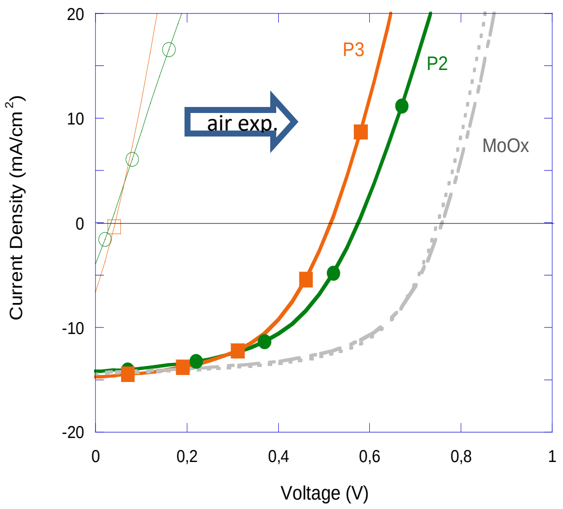


**Figure S18.** J–V curves of inverted geometry PTB7-Th:$PC_{71}BM$ devices prior and after storage under ambient atmosphere (8 h); AILs: **P2** (green, circles), **P3** (orange, squares). Before air exposure: empty dots; after air exposure: full dots; MoOx: (grey lines), prior (dot)/after (dot-line) air exposure.

**Table S4.** Summary of the PV parameters of PTB7-Th:$PC_{71}BM$ devices with inverted geometry, using **P2**, **P3**, and MoOX; prior and after air exposure; average values over six devices.

| AIL | Air exposure (min) | Voc (V) | FF (--) | Jsc (mA/cm²) | PCE (%) |
|---|---|---|---|---|---|
| **P2** | - | 0.012 | 0.07 | 1.67 | 0.02 |
| | 480 | 0.57 | 0.51 | 14.42 | 4.24 |
| **P3** | - | 0.04 | 0.28 | 9.09 | 0.08 |
| | 480 | 0.53 | 0.52 | 14.45 | 4.02 |
| MoOx | - | 0.74 | 0.60 | 14.21 | 6.35 |
| | 480 | 0.76 | 0.60 | 14.1 | 6.36 |

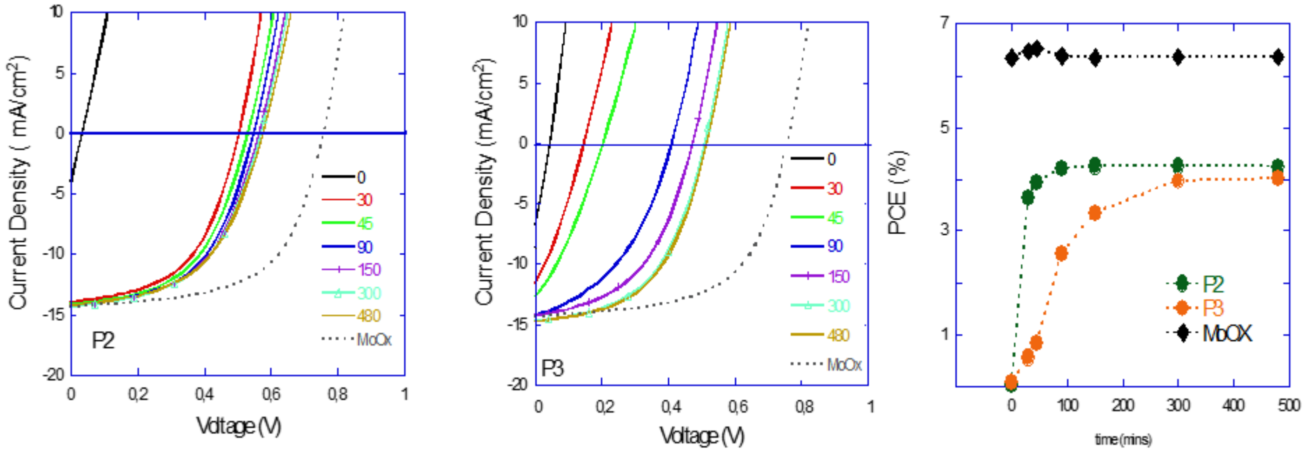


**(a)** **(b)** **(c)**

**Figure S19.** Evolution as a function of time of storage under ambient atmosphere of the J–V curves of PTB7-Th:$PC_{71}BM$ devices with inverted geometry, using **P2** (**a**) and **P3** (**b**) as AIL. (**c**) Evolution of the PCE of the devices using **P2**, **P3** and MoOx AILs.

## References


1. Zotti G.;Berlin A.; Vercelli B.; Electrochemistry of conjugated planar anticancer molecules: Irinotecan and Sunitinib, *Electrochim. Acta* **2017**, 231, 336-343. https://doi.org/10.1016/j.electacta.2017.02.043.
2. Iosip, M. D.; Destri, S.; Pasini, M.; Porzio, W.; Pernstich, K. P.; Batlogg, B. New Dithieno[3,2-b:2′,3′-d]Thiophene Oligomers as Promising Materials for Organic Field-Effect Transistor Applications. *Synth. Met.* **2004**, *146* (3), 251–257. https://doi.org/10.1016/j.synthmet.2004.08.004.
3. Carulli, F.; Scavia, G.; Lassi, E.; Pasini, M.; Galeotti, F.; Brovelli, S. Giovanella, U.; Luzzati, S.A bifunctional polyfluorene combining ammonium and phosphonate functionalities as an efficientcathode interfacial material for polymer solar cells. *J. Colloid Interface Sci.* **2019**, 538, 611-619. https://doi.org/10.1016/j.jcis.2018.12.027
